# Whole-slide deep-learning quantification of fibrosis and pathologist-defined proliferative/neoplastic regions in a thioacetamide-induced rat liver model

**DOI:** 10.3389/frai.2026.1884918

**Published:** 2026-09-25

**Authors:** Feng Liu, Yating Pan, Xinyi Liao, Yating Deng, Deqiang Cheng, Guanzhen Yu, Ying Chen, Yu Cheng

**Affiliations:** 1 School of Information and Control Engineering, China University of Mining and Technology, Xuzhou, China; 2 School of Information Engineering, Yulin University, Shaanxi, Yulin, China; 3 Department of Oncology, Longhua Hospital, Shanghai University of Traditional Chinese Medicine, Shanghai, China; 4 Allergy and Cancer Research Center, Tongji Hospital, School of Medicine, Tongji University, Shanghai, China; 5 Department of Gastroenterology, Changhai Hospital, Naval Medical University, Shanghai, China

**Keywords:** deep learning, fibrosis, hydrogen intervention, liver pathology, proliferative/neoplastic lesion, whole-slide image (WSI)

## Abstract

Automated analysis of whole-slide images may support objective quantification of selected lesions in experimental liver pathology. Using H&E-stained whole-slide images from rats exposed to thioacetamide (TAA), we developed a deep-learning framework for whole-slide segmentation and quantification of two prespecified targets: fibrosis and a single pooled class of pathologist-defined proliferative/neoplastic regions. Benign proliferation, dysplasia/preneoplasia, and malignant neoplasia were not annotated or trained as separate subclasses. The available project archive did not contain an auditable whole-slide- or animal-level training/validation manifest; the available training code implements a patch-level random split. The framework generated fibrosis proportion, proliferative/neoplastic-region proportion, and the count of spatially separated positive regions for each whole-slide image. These model-derived measurements were used to describe changes across experimental time points and to explore differences between the TAA and TAA + H_2_ groups. The TAA + H_2_ group showed a lower model-derived burden of the two selected lesion categories. The framework does not assess the full spectrum of TAA-associated liver pathology and should not be interpreted as an independently validated diagnostic classifier. Independent animal-level validation is required before diagnostic or therapeutic claims can be made.

## 1 Introduction

Scientific integrity is fundamental to biomedical research, and image manipulation can undermine both the reliability of the literature and public confidence in research (Kearney et al., 2024; Weber et al., 2026; Candal-Pedreira et al., 2023; Armond et al., 2024). Although image-processing software has legitimate analytical uses, it can also be misused to alter scientific images (Ding et al., 2024; Sunen et al., 2024). Methods that improve the traceability and whole-slide visualization of pathological findings may therefore help readers evaluate the relationship between representative fields and the complete tissue section (Parashar et al., 2022; Ali et al., 2022). Artificial intelligence (AI)-assisted image analysis is one possible approach to this task (Grignaffini et al., 2024; Hori et al., 2026).

AI refers to a technology that empowers computers to simulate human intelligence. The rapid advancement of machine learning, particularly deep learning, has brought transformative changes to people’s daily lives (Zhang et al., 2025; Wang et al., 2025b). AI harbors tremendous potential across medical applications, ranging from auxiliary clinical diagnosis and patient administration to therapeutic regimen formulation (Hallquist et al., 2025). The sheer volume that characterizes medical data provides room for continuous deep learning advancement (Alhejaily 2025; Akinrinmade et al., 2023). AI is currently driving various imaging-related fields, including radiology (Guarnera et al., 2026), oncology (Wang et al., 2026) and digital pathology (Zhang et al., 2024).

AI has been shown to be capable of automatically detecting tumor tissues, thus reducing pathologist workloads (Erion Barner et al., 2023). In fact, AI can extract more histological features required for tumor staging prediction than human pathologists, a capability that is particularly valuable for gastrointestinal and hepatic tumors (El-Khoury and Zaatari 2025). Studies have shown that the presence of tumor-infiltrating lymphocytes is closely related to the prognosis of gastric cancer patients and that tumor-infiltrating lymphocytes can be detected in pathological tissues using Convolutional Neural Network (CNN) models—providing strong predictive prospects for tumor prognosis (Yoshida and Kiyuna 2021; Xie et al., 2022). In hepatocellular carcinoma (HCC), AI models have been shown to predict post-resection survival more accurately than multifaceted composite total scores (Seven et al., 2025). Overall, AI can infer molecular and genetic changes in cancer tissues from digital histological sections (Zhang et al., 2026). Taken together, this evidence suggests that the application of AI in pathology may represent an opportunity for developing a precision treatment for digestive system tumors.

Liver lesions reflect changes in the organ’s function and metabolism and can be caused by external stimuli or by local disease (Fan et al., 2024; Yang et al., 2024). By observing morphological and pathological changes in the liver, we can understand the onset and progression of various diseases, as well as the body’s response to drug efficacy and toxicity (Gan et al., 2025). The majority of liver lesions in animal models arise from inflammatory diseases (Taru et al., 2024), metabolic diseases (Westcott et al., 2024), and proliferative diseases (Qiu et al., 2021). In previous studies, we have established that thioacetamide (TAA) induced abnormal proliferation in the epithelium of the intrahepatic bile duct epithelium can lead to neoplasia (Li et al., 2022). We saw positive results from interventions involving aspirin, herbs, and hydrogen-rich water (El Sadda et al., 2023; Wang et al., 2025a; Mai et al., 2020; Wu et al., 2024; Li et al., 2021). In the experiment involving the hydrogen-rich water intervention, our algorithmic model clearly indicated both the location and extent of liver fibrosis, visualized the differences between the two treatment options, and provided objective quantitative indicators (Li et al., 2021). However, objective differentiation and whole-slide quantification of fibrosis and proliferative/neoplastic lesions remained incompletely resolved.

Therefore, this study aimed to develop a whole-slide image-analysis framework for two selected histopathological targets in a TAA-induced rat liver model: fibrosis and a pooled class of pathologist-defined proliferative/neoplastic regions. This framework seeks to reconstruct plaque-level prediction outcomes using the original slice coordinates and to generate measurements based on both regions and connected regions. Furthermore, it lacks the capability to identify uneven, serrated, or wavy liver margins, as well as to visualize the multi-stage pathological progression of cholangiocarcinoma. Tissue-boundary detection was used only to define the analysis mask. The study did not systematically evaluate other TAA-associated changes, did not classify benign, preneoplastic, and malignant lesions as separate categories, and was not designed to establish diagnostic accuracy or treatment efficacy.

## 2 Methods

### 2.1 Pathology data sets

The manuscript dataset was described as 56 H&E-stained rat liver whole-slide images (WSIs): 23 TAA slides, 22 TAA + H_2_ slides, and 11 normal liver slides. A historical slide-selection file identifies the 23 TAA and 22 TAA + H_2_ slide IDs, which could be matched to source-image and output files; it does not identify the 11 normal slides (Figure 1; Supplementary Figure S1). The available training code constructs a dataset of cropped patches and applies random_split to those patches; no archived WSI-level or animal-level partition manifest was found. We therefore cannot verify the reported 42/14 WSI split, the claim that one WSI contributed patches to only one partition, the number of unique animals, or possible cross-partition animal overlap. The reported validation must be described as an unverified historical internal validation until the original split manifest and animal-to-slide linkage table are supplied.

**Figure 1:**
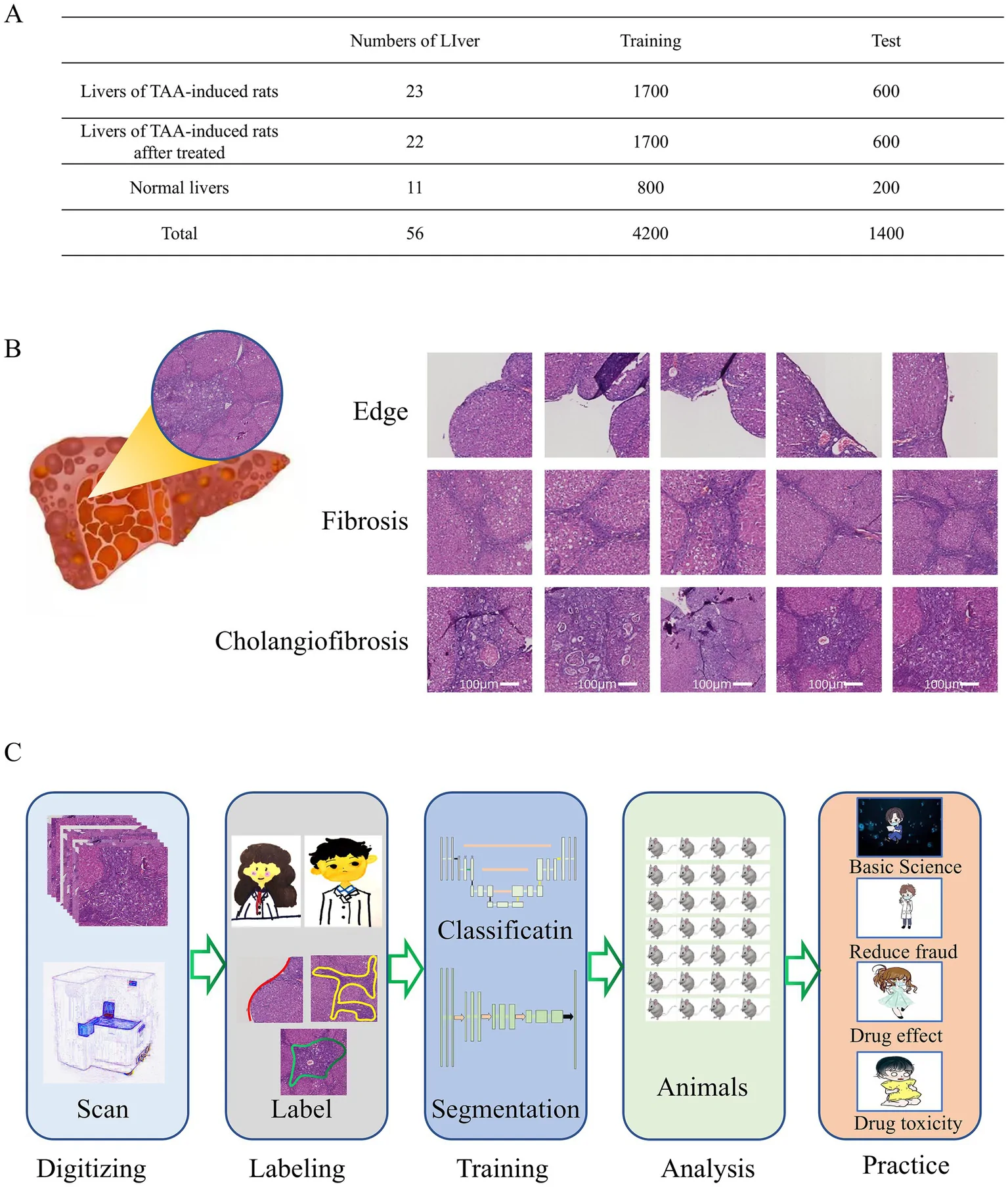
Pathology dataset and deep learning network analysis flowchart for liver disease segmentation. **(A)** Pathology dataset used in this model. The training set was composed of 4,200 WSIs, and the test set was composed of 1,400 WSIs. **(B)** The main recognition components of this model, including liver edge, liver fibrosis, and regions of cholangiofibrosis. **(C)** Flowchart showing model’s entire operational analysis. There are five steps, including digital scanning of pathological slides, manual labeling, classification and cutting of the training model, result analysis, and testing practice.

Pathological annotation was reported to have been performed in ASAP by three pathologists who were blinded to experimental group allocation. Two target masks were available in the project archive: fibrosis and a single pooled proliferative/neoplastic category. Fibrosis was defined by expansion of portal or peribiliary fibrous stroma and/or the presence of fibrous septa or bridging bands. The second target was defined by atypical biliary or epithelial proliferation with irregular structures and associated stromal reaction. Benign proliferation, dysplasia/preneoplasia, and malignant neoplasia were not annotated or trained as separate subclasses; therefore, the model cannot distinguish among these entities. The analysis files supplied for this revision did not contain a signed annotation standard operating procedure, versioned ASAP project, or record specifying how every mixed region was resolved. We therefore cannot retrospectively verify whether non-fibrotic inflammatory regions, necrosis, hemorrhage, hepatocellular vacuolation, or other non-target changes were represented as background/negative pixels in every sampled patch. This point must be verified against the original annotation protocol before resubmission. All reference annotations were based on H&E sections; no collagen-specific stain or immunohistochemical confirmation was available. Figure 1 shows calibrated representative H&E fields from an original NDPI source and their coordinate-matched available binary reference masks. All sections were H&E stained. Representative H&E examples and corresponding masks are provided in Figures 1B, 2A–C.

**Figure 2:**
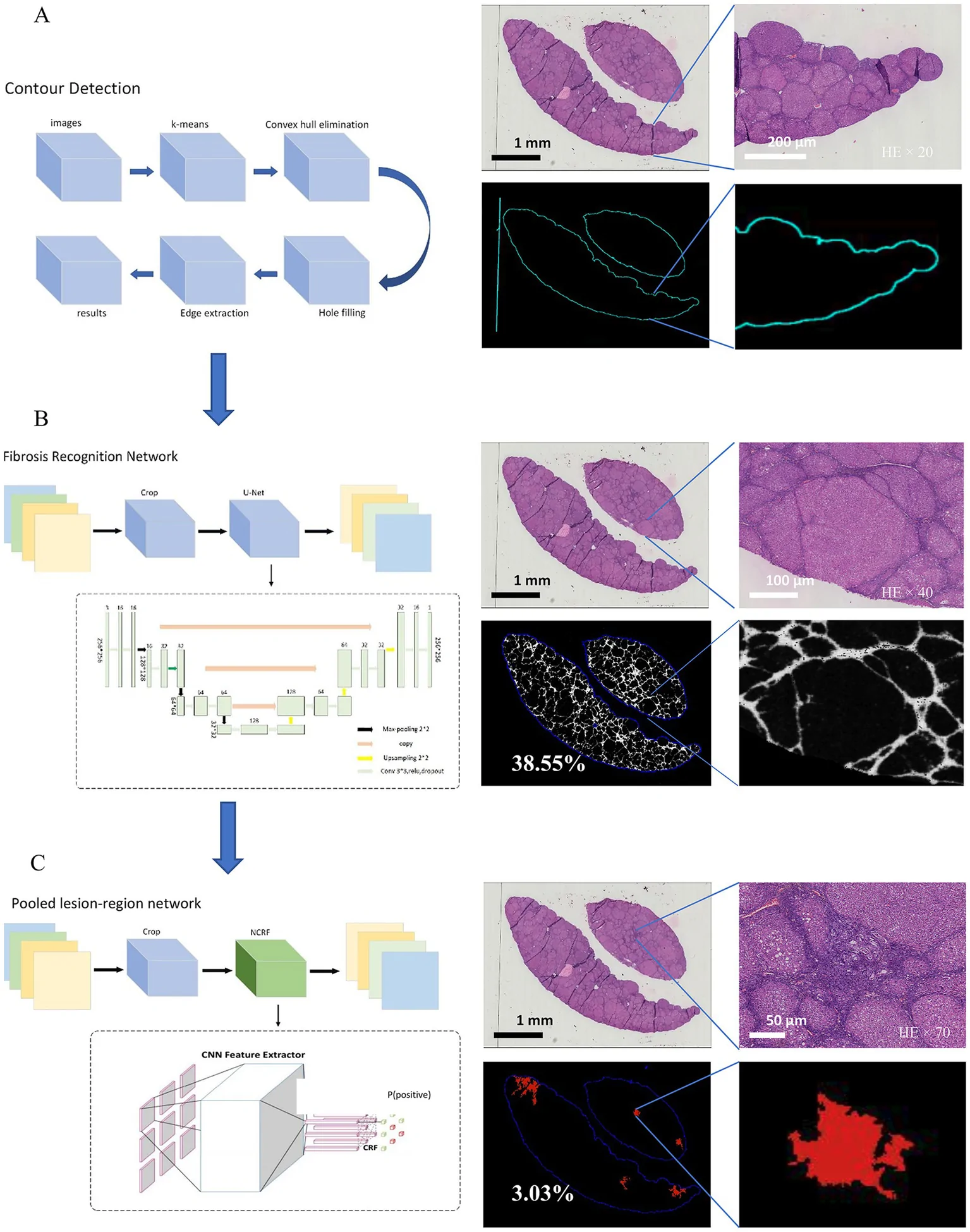
Workflows for tissue-region detection and segmentation of the two selected lesion categories. **(A)** Tissue-region detection used to define the analysis mask; liver-margin morphology was not an analyzed endpoint (blue sections are identified liver edges). **(B)** U-Net fibrosis segmentation with representative H&E fields and predicted masks (white sections are the identified fibrosis areas). **(C)** NCRF segmentation of the pooled pathologist-defined proliferative/neoplastic lesion-region class with representative H&E fields and predicted masks. The pooled class was not subdivided into benign, preneoplastic, and malignant lesions (red sections are identified tumor areas).

### 2.2 Tissue-region detection

We first identified the tissue region in each WSI. K-means clustering was used to obtain an initial region of interest, followed by convex-hull processing and hole filling to generate the tissue mask (Figure 2A). This mask defined the denominator for downstream area measurements. Liver-margin morphology was not annotated, quantified, used as a classifier feature, or analyzed as an independent biological endpoint.

### 2.3 Identification of tissue fibrosis

Fibrosis was identified within the tissue-region mask using the workflow shown in Figure 2B. The fibrosis annotations generated in ASAP served as the reference masks. Patches containing fibrosis were preferentially sampled to improve representation of the positive class, while their original WSI coordinates were retained for whole-slide reconstruction. The detailed handling of non-fibrotic inflammatory tissue and other mixed changes within sampled patches must be verified against the original annotation protocol before resubmission.

Because U-Net architectures have performed well in medical-image segmentation, we used a U-Net for end-to-end fibrosis segmentation. Regions of interest were cropped from the source images, resized to 256 × 256 pixels, and normalized. The model used cross-entropy loss, a learning rate of 0.0001, a maximum training step setting of 100, a down sampling rate of 0.5, and the Adam optimizer. Rotation and mirroring were used for data augmentation. During inference, the model generated a binary fibrosis mask for each patch. Patch masks were mapped to their original WSI coordinates and combined. Fibrosis proportion was calculated as predicted fibrosis-mask area divided by total tissue-mask area, multiplied by 100.

### 2.4 Tumor identification

The pooled proliferative/neoplastic lesion-region task was performed after tissue-region and fibrosis processing (Figure 2C). Pathologist-defined positive regions were annotated in ASAP. Because positive regions were relatively sparse, patches containing these annotations were preferentially sampled for training, while the original WSI coordinates were retained for reconstruction. The positive class was not subdivided into benign proliferation, dysplasia/preneoplasia, or malignant neoplasia.

We used the Neural Conditional Random Field (NCRF) framework for patch-based segmentation of the pooled proliferative/neoplastic target. Unlike a conventional convolutional neural network that predicts each patch independently, NCRF first encodes each spatially ordered patch and then models relationships among neighboring patches on a dense grid. The conditional random-field component regularizes neighboring predictions, reducing isolated positive regions and producing a more spatially coherent lesion-region mask (Figure 2C).

During inference, patch-level probabilities were returned to their original WSI coordinates and combined. Proliferative/neoplastic-region proportion was calculated as the reconstructed positive-mask area divided by the total tissue-mask area, multiplied by 100. The positive-region count was obtained by counting spatially separated connected components in the reconstructed mask. These quantities describe correspondence to the pooled pathologist annotation class; they are not independent diagnoses of tumor type or biological behavior. Patches were computational units and were not treated as independent biological observations.

### 2.5 Statistical data

Normality was assessed using the Shapiro–Wilk test, and homogeneity of variance was assessed using Levene’s test. Independent-samples Student’s t-tests were used for the reported between-group comparisons, with *p* < 0.05 considered statistically significant. The study reports historical model-derived lesion burden and exploratory slide-level comparisons. Because the archived code and files do not establish an auditable WSI-level or animal-level partition, the validation results cannot be interpreted as an independent slide-level validation. Sensitivity, specificity, positive predictive value, negative predictive value, calibration, and agreement against an independent animal-level reference cohort were not available and were not reported.

## 3 Results

Whole-slide analysis comprised tissue-region detection, fibrosis segmentation, and segmentation of the pooled pathologist-defined proliferative/neoplastic lesion-region class. Tissue-region detection defined the analysis mask and was not evaluated as a biological liver-margin endpoint. The task-specific processing workflows are shown in Figure 2.

### 3.1 TAA lesion liver

The TAA lesions were divided into 8, 12, 16, and 20-week groups according to lesion cycles. Figure 3A shows the recognition results of different cycles. In order to intuitively understand the influence of lesion cycles on the liver lesions, we calculated the average proportion of the combined proliferative/neoplastic lesion area masks in the pathological sections of TAA liver disease foci at different disease course stages (Figures 3B–D). We found that the mean proportions of liver fibrosis in TAA lesions at 8, 12, 16 and 20 weeks were 2.05 ± 0.11, 7.29 ± 5.80, 29.2 ± 11.54 and 31.44 ± 8.03, respectively. The mean proportions of the pooled proliferative/neoplastic lesion-region mask were 0 ± 0, 0.01 ± 0.02, 1.83 ± 1.21 and 3.93 ± 2.09, and the mean positive-region counts were 0 ± 0, 0.25 ± 0.43, 5.25 ± 2.68 and 4 ± 2.05, respectively. These model-derived measurements increased across the examined time points, but they do not distinguish benign, preneoplastic, and malignant subclasses.

**Figure 3:**
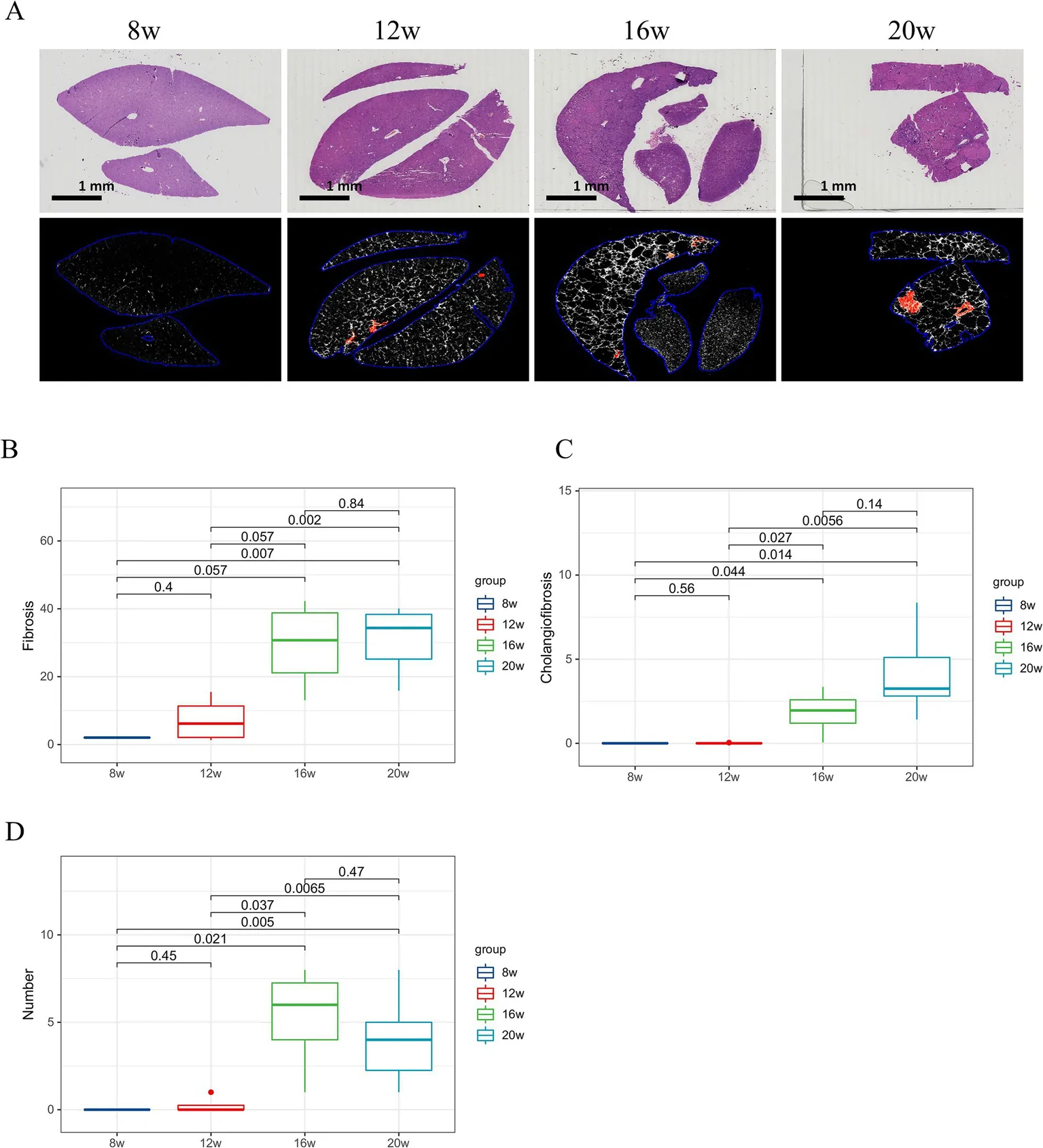
Model-derived measurements in TAA-exposed liver slides across experimental time points. **(A)** Model-derived measurements in TAA-exposed liver slides across experimental time points. **(B)** Percentage of liver fibrosis. **(C)** Proportion of the pooled proliferative/neoplastic lesion-region mask (labeled ‘cholangiofibrosis’ in the original plot). **(D)** Count of spatially separated positive regions. Values are model-derived outputs and do not represent subclass-specific tumor diagnoses. Independent-samples *t*-tests were used for the displayed comparisons. *p < 0.05.

### 3.2 TAA + H_2_ lesions in liver

The TAA + H_2_ lesions were also divided into 8, 12, 16, and 20-week groups according to lesion cycles. Figure 4A shows the recognition results of different cycles. Similar to the liver with TAA lesions, we statistically analyzed the average proportion of the combined proliferative/neoplastic lesion area masks in the liver tissue sections of pathological TAA + H_2_ lesions with different lesion cycles. We then took their average values (Figures 4B–D). The mean proportions of liver fibrosis in TAA lesions at 8, 12, 16 and 20 weeks were 2.35 ± 0.88, 2.29 ± 0.67, 20.07 ± 16.51 and 14.75 ± 16.14, respectively. The mean proportions of the pooled proliferative/neoplastic lesion-region mask were 0 ± 0, 0 ± 0, 0.89 ± 0.95 and 2.44 ± 4.04, respectively; and the mean positive-region counts were 0 ± 0, 0 ± 0, 0.75 ± 0.83 and 1.36 ± 2.14, respectively. These values are model-derived measurements of the pooled annotation class rather than tumor-specific diagnostic results.

**Figure 4:**
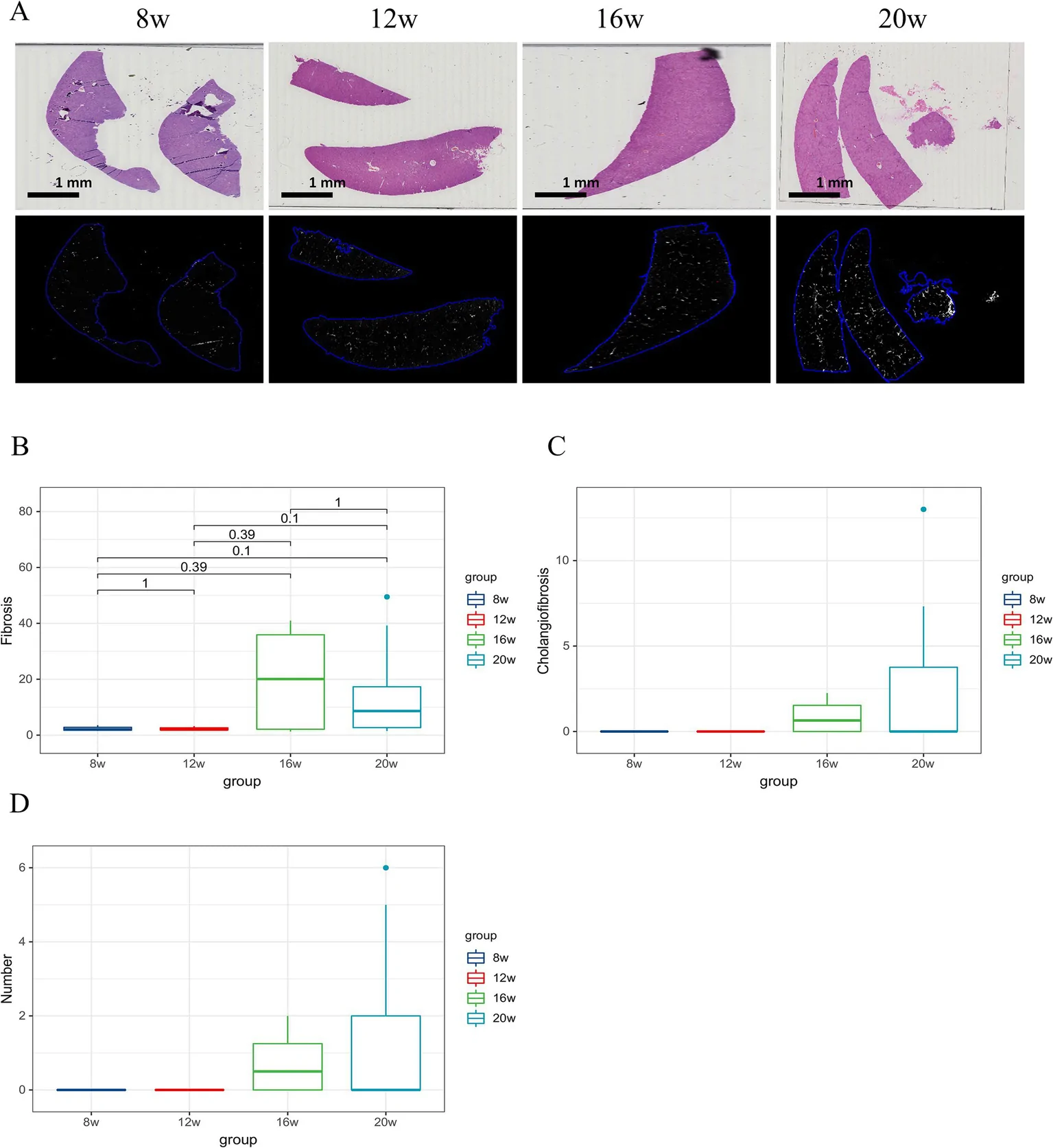
Model-derived measurements in TAA + H_2_ liver slides across experimental time points. **(A)** Representative H&E images and reconstructed model masks at 8, 12, 16, and 20 weeks. **(B)** Percentage of liver fibrosis. **(C)** Proportion of the pooled proliferative/neoplastic lesion-region mask (labeled ‘cholangiofibrosis’ in the original plot). **(D)** Count of spatially separated positive regions. Values are model-derived outputs and do not represent subclass-specific tumor diagnoses. Independent-samples t-tests were used for the displayed comparisons. *p < 0.05.

### 3.3 Comparison of TAA and TAA + H_2_ lesions in liver

In order to verify the effect of H_2_ on the liver with TAA lesions, We compared fibrosis proportion, pooled proliferative/neoplastic lesion-region proportion, and positive-region count between the TAA and TAA + H_2_ groups (Figure 5A), the liver fibrosis from TAA + H_2_ lesions was significantly lower than that in TAA-lesioned liver samples. Comparative statistical figures are shown in Figures 5B–D. Across the examined time points, the TAA + H_2_ group generally showed lower model-derived values than the TAA group, although the magnitude and variability differed by time point and outcome. These exploratory slide-level comparisons describe treatment-associated histological differences in the two selected annotation classes. They do not establish therapeutic efficacy and require confirmation in an independent animal-level design.

**Figure 5:**
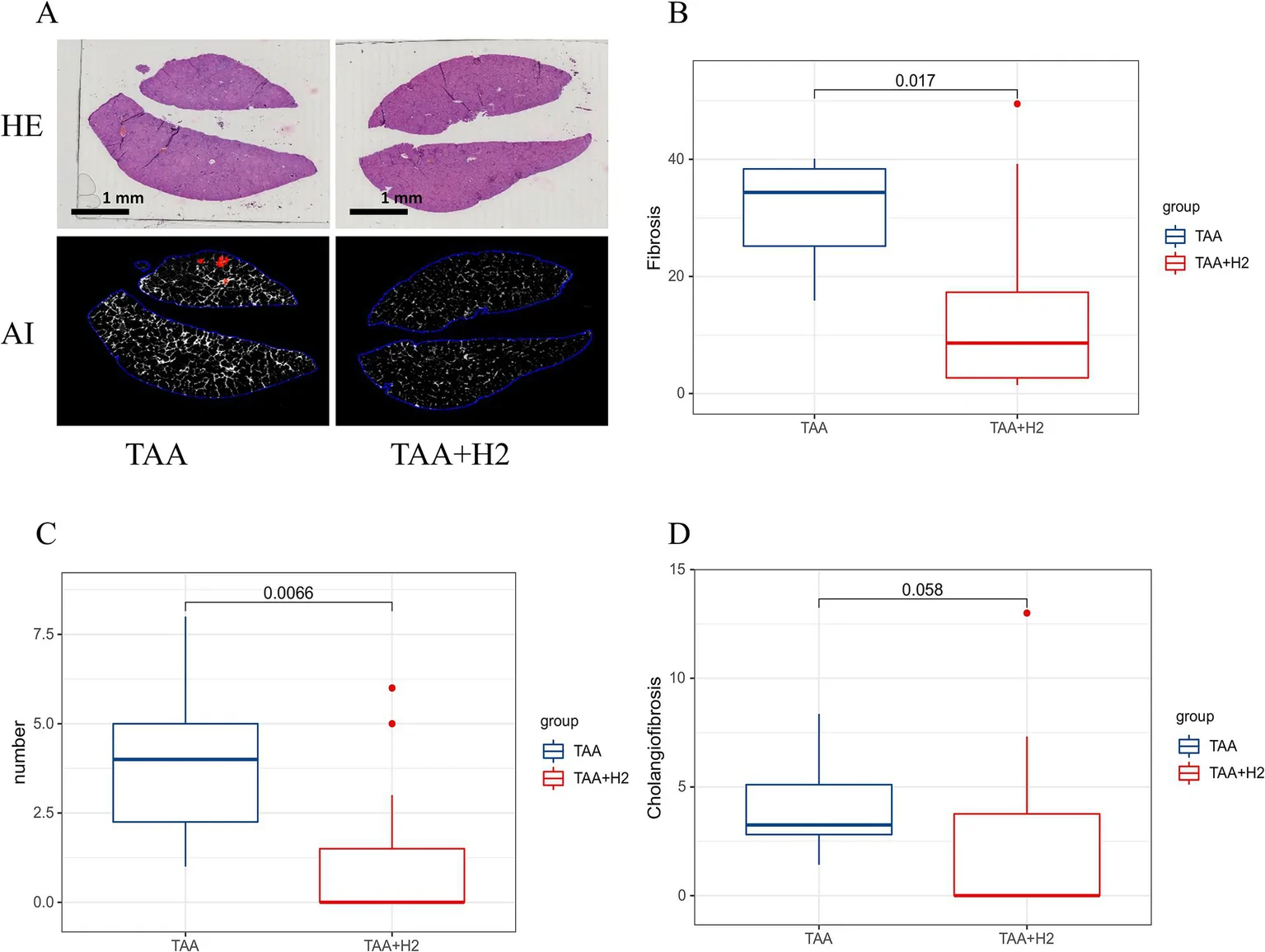
Exploratory comparison of model-derived lesion measurements between TAA and TAA + H_2_ slides. **(A)** Representative H&E images and reconstructed masks. **(B)** Percent of liver fibrosis. **(C)** Count of spatially separated regions positive for the pooled proliferative/neoplastic annotation class. **(D)** Proportion of the pooled proliferative/neoplastic lesion-region mask. These slide-level comparisons do not establish tumor-specific diagnostic accuracy or therapeutic efficacy. Independent-samples *t*-tests were used for the displayed comparisons. *p < 0.05.

## 4 Discussion

This study demonstrates the feasibility of using a deep-learning workflow to reconstruct and quantify two selected pathologist-defined targets across rat liver WSIs. The model segments fibrosis and a pooled proliferative/neoplastic lesion-region class and then derives area proportions and connected-region counts from the reconstructed masks. Tissue-boundary detection is used only to define the analysis mask; liver-margin morphology is outside the evaluated endpoints. Likewise, correspondence between model masks and the pooled reference annotations should not be described as independently established diagnostic accuracy. This study did not quantitatively assess the irregularity of the liver margin as a biological endpoint. Consequently, the tumor output value should be interpreted as the segmentation result of the proliferative or neoplastic region as defined by pathologists, rather than as an independently validated diagnostic classification system.

TAA-associated liver sections may contain fibrosis, necrosis, hemorrhage, inflammatory infiltrates, hepatocellular vacuolation, proliferative changes, and normal parenchyma within the same WSI. The model outputs were represented as two distinct binary targets, fibrosis and the pooled proliferative/neoplastic category. However, the supplied files did not include a verifiable rule for resolving every mixed region or for representing non-fibrotic inflammation and other non-target changes during training. Consequently, the historical workflow cannot be used to establish whether these changes served as negative/background pixels in every sampled patch. This limitation should be resolved by reviewing the original annotation protocol and project files before resubmission; any confirmed rule should then be reported explicitly.

Cancer develops through a multi-step process of somatic cell evolution accompanied by a multi-step morphological continuum of transformation from normal cells to preneoplastic, benign, and then malignant tumors (de Visser and Joyce 2023; Dixit et al., 2026). Mutagenic forces lead to a time-dependent accumulation of point mutations throughout the cancer transformation process, which can be observed through the progressively deteriorating morphology of healthy tissue (Gerstung et al., 2020). This morphological and pathological changes (Jang et al., 2021), along with specific driver mutations (Panigrahi et al., 2023), can be identified and extracted using machine learning methods. Not surprisingly, automated histopathological analysis depends on image features--including cell size, shape, distribution of pixel intensities, the texture of cells and nuclei, and tumor microenvironment (Venturi et al., 2025). The multi-step CCA process would go through a normal bile duct state, followed by biliary dysplasia and infiltrative CCA. This process can be pathologically reproduced in TAA-induced rat models (Massa et al., 2020).

In the present study, these features were used to distinguish the pathologist-defined target categories of fibrosis and proliferative/neoplastic lesions in the TAA-induced rat model. The reconstructed WSI masks allow the spatial distribution and burden of these categories to be documented systematically. Because the reference annotations were based on H&E morphology and no immunohistochemical confirmation or independent animal-level test cohort was available, the output should be considered a quantitative research measure rather than a definitive diagnosis of cholangiocarcinoma.

Machine learning techniques have proven useful in predicting drug responses and synergism (El Khili et al., 2023), as well as adverse drug reactions (Chen et al., 2022). For example, researchers have used cellular isolation methods to reflect the extent of drug effects on cells based on the cellular characteristics of cyclic drug administration (Kim and Lee 2025), and other groups have developed an automated machine learning (AutoML) model to classify patterns of drug-induced liver injury over WSIs (Puri 2020). We previously developed an automated lesion detection framework, Alesion, to detect liver lesions from WSIs. This AI algorithm can observe the location and number of liver lesions in WSIs, and clearly show treatment effects of H_2_ compared to controls (Li et al., 2021). A limitation of this AI algorithm is that it does not distinguish between the different pathological steps of biliary dysplasia and infiltrative CCA. In the present study, we extended this approach to separately quantify fibrosis and pathologist-defined proliferative/neoplastic lesions. Across the experimental time points, the model-derived lesion burden was lower in the TAA + H_2_ group than in the TAA group, supporting the use of the framework for exploratory comparison of treatment-associated histological changes. Our research results also indicate that this model can be used to evaluate the protective effect of hydrogen-rich saline on liver injury in another animal model (Supplementary Figure S1) (Zhang et al., 2018). These observations should not be interpreted as a validated estimate of therapeutic efficacy until confirmed with independent animal-level samples and prespecified diagnostic-performance and agreement analyses.

Scientific misconduct is a global problem in health-related research. Misconduct can be classified into four categories: overall concerns, textual concerns, image concerns, and data concerns (Candal-Pedreira et al., 2023; Bordewijk et al., 2021). However, the tools to investigate all of these kinds of research misconduct are rudimentary and labor-intensive (Tang et al., 2025). One of the four types of research misconduct is pathological image misconduct. In previous studies, pathology results were presented as small slices with specific magnifications of HE or IHC rather than WSIs of organs or tumor tissues. Fraudulent behavior, including selecting only areas of interest, ignoring areas without lesions, or even taking representative photographs from different parts of the same sample, can occur as a result of this process (Dal-Ré and Marušić 2025). We need automated tools to address these rising challenges. The best way to do this is to present all lesions in WSIs in a way that can be easily understood by laypeople. Despite the visualization and quantification of liver lesions in Supplementary Figure S1, it is still difficult to distinguish whether these images are from different samples or from the same sample. However, AI-based automatic classifiers for WSIs could effectively avoid this type of scientific misconduct.

This study has several limitations. First, the reference standard was based on H&E morphology without collagen-specific staining or immunohistochemical confirmation. Second, the proliferative/neoplastic target was a pooled category and did not distinguish benign proliferation, preneoplasia, and malignancy. Third, the supplied files did not document a verifiable rule for mixed lesions or confirm whether non-fibrotic inflammatory regions were used as negative/background pixels during training. Fourth, although source images were recovered for the 23 TAA and 22 TAA + H_2_ slides, the archive did not identify the 11 normal slides described in the manuscript and did not contain a verified animal-to-slide linkage table. Fifth, the available training code applies a patch-level random split, while no WSI-level or animal-level partition manifest was found; therefore, the previously stated WSI-level 42/14 partition cannot be verified and the historical validation may be affected by patch, slide, or animal correlation. Sixth, no independent animal-level test cohort or prespecified diagnostic-performance and pathologist-model agreement analysis was available. Finally, calibrated high-resolution representative H&E fields with coordinate-matched available binary masks were recovered for Supplementary Figure S2, but their use as evidence of the original annotation protocol still requires confirmation against the original versioned pathology record.

## 5 Conclusion

In this study, we developed a WSI-based deep-learning framework to quantify fibrosis and a single pooled class of pathologist-defined proliferative/neoplastic regions in a TAA-induced rat liver model. The framework generated whole-slide area proportions and connected positive-region counts and was used for exploratory comparison of TAA and TAA + H_2_ slides. The model does not evaluate the full spectrum of TAA-associated pathology, distinguish benign from preneoplastic or malignant proliferative lesions, or provide independently validated diagnostic or therapeutic conclusions.

## Data Availability

The original contributions presented in the study are included in the article/Supplementary material, further inquiries can be directed to the corresponding authors.
